# ALDH1A3–Linc00284 Axis Mediates the Invasion of Colorectal Cancer by Targeting TGF*β* Signaling via Sponging miR-361-5p

**DOI:** 10.1155/2022/6561047

**Published:** 2022-10-13

**Authors:** Chunlin Ke, Minmin Shen, Peirong Wang, Zhihua Chen, Suyong Lin, Feng Dong

**Affiliations:** ^1^Department of Radiotherapy, Cancer Center, The First Affiliated Hospital of Fujian Medical University, Fuzhou, Fujian 350000, China; ^2^Key Laboratory of Radiation Biology of Fujian Higher Education Institutions, The First Affiliated Hospital, Fujian Medical University, Fuzhou, Fujian 350000, China; ^3^Department of Gastrointestinal Surgery, The First Affiliated Hospital of Fujian Medical University, Fuzhou, Fujian 350000, China

## Abstract

ALDH1A3 and Linc00284 involve in colorectal cancer (CRC) development; however, the regulatory mechanism is still unclear. In this study, we collected clinicopathological characteristics and tissue samples from 73 CRC patients to analyze the expression of ALDH1A3, Linc00284, TGF*β* signaling and miR-361-5p using qPCR, Western blotting, and ELISA. Multiple CRC cell lines were evaluated in this study, and the highest level of ALDH1A3 was observed in SW480 cells. To investigate the regulatory mechanism, RIP and luciferase assays were used to validate the interaction between Linc00284, miR-361-5p, and TGF*β*. Proliferation, viability, migration, and invasion assays were performed to profile the effects of the ALDH1A3–Linc00284 axis in CRC cell functions, which was upregulated in CRC tissues. Knockdown ALDH1A3 or Linc00284 significantly reduced TGF*β* expression and suppressed the EMT process, while overexpression had opposite effects. miR-361-5p targeted TGF*β* directly, which negatively correlated with ALDH1A3–Linc00284 expression and CRC progression. Mechanistically, upregulation of ALDH1A3–Linc00284 promotes colorectal cancer invasion and migration by regulating miR-361-5p/TGF*β* signaling pathway. Dysregulation of the ALDH1A3–Linc00284-miR-361-5p-TGF*β* axis causes CRC invasion, which might provide a new insight into the treatment of CRC.

## 1. Introduction

Colorectal cancer is the second highest mortality tumor worldwide [1, 2]. By 2030, the number of global cases of CRC is expected to increase by 60%, with more than 2.25 million new cases and 1.15 million deaths from CRC cancer each year [3, 4]. In the past few decades, CRC has become one of the most common cancers, the incidence has increased from 1.0% annually to 2.4% since 1974 in the USA [5]. In most Europe, the incidence of CRC is increasing every year, ranging from 0.4% to 3.6% [6]. The existing therapies for CRC include surgery, radiotherapy, chemotherapy, targeted drug therapy, and so on. Immunotherapy is an encouraging weapon to treat CRC, but so far it has only succeeded in a small proportion of CRC [7]. The prognosis of metastatic CRC is worse, with an overall survival time of only about 30 months [8]. Therefore, it is very urgent to develop new effective strategies to control CRC, especially metastatic CRC.

Cumulative evidence indicates that ALDH1A3 and Linc00284 play an important role in the progression of CRC [9–12]. ALDH1A3 is a known marker of cancer stem cells which has been shown to be important for the proliferation, migration, and maintenance of the mesenchymal cancer stem cell phenotype [13]. Durinikova et al. demonstrated that ALDH1A3 was increased in CRC tissues, which promotes spontaneous metastasis formation and associates with acquired chemoresistance of colorectal cancer [9]. Long noncoding RNAs (lncRNAs) Linc00284 has been reported to be involved in the initiation and progression of many cancers, including oral squamous cell carcinoma, ovarian carcinoma, papillary thyroid cancer, lung cancer, CRC, and so on [12, 14–17]. Our recent study demonstrated that Linc00284 exhibits oncogenic function and promotes the progression of CRC through upregulating the expression of c-Met [12]. However, the underlying molecular mechanisms of ALDH1A3 and Linc00284 on the growth and metastasis of CRC are still unclear. Here, we investigated the role of the ALDH1A3–Linc00284 signal in CRC progression and in the tumor microenvironment through in-depth analysis of clinical data and in vitro experiments. This study proves that ALDH1A3–Linc00284 upregulates TGF*β* signaling through miR-361-5p, and then promotes the epithelial–mesenchymal transition (EMT) process and CRC tumor metastasis. This research will provide a key theoretical basis for the CRC treatment and drug development.

## 2. Materials and Methods

### 2.1. Human Study

CRC tissues and the paired adjacent nontumor samples were derived from 73 CRC patients, which are consistent with the patients and samples in our previous study [12], including the patient's information, inclusion and exclusion criteria, and metastatic and relapse records. The TNM stages of CRC in the above patients were classified based on the American Joint Committee on Cancer (AJCC) tumor, lymph node, metastasis (TNM) system. The study was obtained the written informed consent from all participants and approved by the ethics committee of the First Affiliated Hospital of Fujian Medical University.

### 2.2. Cell Culture

HEK293T cell lines and CRC cell lines, SW480, HCT116, LS174T, DLD-1, HCT15, and SW620, were ordered from ATCC (Rockville, MD) and used in this study. HEK293T, SW480, HCT116, SW620, and LS174T cells were cultured in DMEM supplemented with 100 U/ml penicillin/streptomycin (Gibco, Grand Island, NY) and 10% fetal bovine serum (Gibco). DLD-1 and HCT15 cells were cultured in RPMI-1640 supplemented with100 U/ml penicillin/streptomycin (Gibco) and 10% fetal bovine serum (Gibco). Cell cultures were placed in cell culture incubator with 95% humidity and 5% CO_2_ at 37°C.

### 2.3. Construction and Transfection

The small hairpin RNA (shRNA) of ALDH1A3 was purchased from Sigma-Aldrich (TRCN0000027144, TRCN0000027160, TRCN0000027183, St. Louis). The primers for shRNA chains sequences were: TRCN0000027144: forward, 5'-CCGGGCTG TATTAGAACCCTCAGATCTCGAGAAATTGTGTCTGAAGAGAATGTTTTTG-3', reverse, 5'-AATTCAAAAACATTCTCTTCAGACACAATTTCTCGAGATCTGAGGGT TCTAATACAGC-3'; TRCN0000027160: forward, 5'-CCGGGAGCAGGTCTACTCTGA GTTTCTCGAGAAATTGTGTCTGAAGAGAATGTTTTTG-3', reverse, 5'-AATTCAA AAACATTCTCTTCAGACACAATTTCTCGAGAAACTCAGAGTAGACCTGCTC-3'; TRCN0000027183: forward, 5'-CCGGGCCGAATACACAGAAGTGAAACTCGAGAA ATTGTGTCTGAAGAGAATGTTTTTG-3', reverse, 5'-AATTCAAAAACATTCTCTTC AGACACAATTTCTCGAGTTTCACTTCTGTGTATTCGGC-3'. Linc00284 shRNA, mimic and inhibitor of miR-361-5p, full-length of ALDH1A3 and Linc00284, and negative controls were ordered from Genechem (Shanghai, China). The stable ALDH1A3 knockdown and Linc00284 knockdown SW480 cells were established by using the corresponding lentivirus generated from HEK293T cells as described previously [12]. Lentivirus expression and package vectors, pLVX pVSVG, pRSV-REV, and pMDLg/pRRE were purchased from Genechem. The pcDNA3.1 vector was used to construct the overexpression plasmid of ALDH1A3 and Linc00284. Lipofectamine™ 2000 Transfection Reagent (Thermo Fisher Scientific Inc, Waltham, MA) for cell transfection following the manufacturer's instructions.

### 2.4. qRT-PCR

Total RNA from homogenize tissue samples and lysed cells was extracted using Trizol reagent (Invitrogen, Waltham, MA) according to the protocol provided by the manufacturer. TaqMan® Reverse Transcription Reagents and random primers were used to synthesize the cDNA. StepOne™ PCR System (Thermo Fisher Scientific Inc) was used to perform qPCR reaction. The expression of target genes was normalized by GAPDH or U6. All primers used in this study were listed in Table 1.

### 2.5. Western Blotting

Proteins were extracted from homogenized tissue samples by using RIPA buffer containing PMSF and protease inhibitor cocktails (KeyGEN BioTECH, Nanjing, China). Protein levels of ALDH1A3 and *β*-Actin were determined using Western blotting as described previously [12]. The primary antibodies and information were listed below: *β*-Actin (Proteintech, 60008-1-Ig, 1 : 2000), ALDH1A3 (Novus, NBP2-46510, 1 : 1000), E-cadherin (Cell Signaling Technology, 3195, 1 : 2000), N-cadherin (Cell Signaling Technology, 13116, 1 : 2000), Vimentin (Cell Signaling Technology, 5741, 1 : 2000).

### 2.6. Elisa

The protein level of TGF*β* in Linc00284- and ALDH1A3-overexpressed SW480 cells medium was determined by ELISA (DB100B, R&D Systems, Minneapolis, MN) according to the manufacturer's instructions.

### 2.7. RNA Immunoprecipitation

RNA Immunoprecipitation (RIP) assay was performed to detect the interaction between Linc00284 and miR-361-5p by using the Magnetic RNA-Protein Pull-Down Kit (Pierce, Waltham, MA). In brief, SW480 cells were lysed in RIP buffer firstly, and then incubated with Argonaute2 (Ago2) antibody beads (Sigma-Aldrich) overnight at 4°C, anti-mouse IgG magnetic beads were used as negative control. The RNA that bound to beads was extracted and detected by RT-qPCR.

### 2.8. Luciferase Assay

The TGF*β* 3'-UTR WT, Linc00284 WT, and the corresponding mutated fragments were cloned into an overexpression vector (pcDNA3.1). The above constructs or control vectors were co-transfected with the luciferase reporter into HEK293T cells using Lipofectamine™ 2000 (Invitrogen). Two days after transfection, HEK293T cells were lysed and luciferase activity was determined by the Dual-Luciferase® Reporter (DLR™) Assay System (Promega) following the manufacturer's instruction.

### 2.9. Viability and Proliferation Assays

Cell viability of ALDH1A3- or Linc00284-silenced SW480 cells (placed in 96-well plates) at indicated days was evaluated by CCK-8 assay (ab228554, Abcam) following the manufacturer's instructions. Cell proliferation of transfected SW480 cells was determined by Attune Flow Cytometers (Thermo Fisher Scientific Inc)gating with the Ki67 antibody (ab92742, Abcam, 1  : 100 dilution) as described previously [18].

### 2.10. Migration and Invasion Assays

To analyze cell migration, 5 × 10^6^ SW480 cells were seeded in 6-well culture plate. The cell monolayer was scratched by a sterile tip when they reached 90% confluence. The wound healing rate was imaged and calculated every 24 hours. CRC cell invasion was performed by trans-well assay as described previously [12].

### 2.11. Statistical Analysis

SPSS 22.0 statistical software (SPSS Inc.) was used to analyze the data in this study. A two-sided unpaired *t* test was used to compare differences between the two groups. One-way analysis of variance (ANOVA) and post-hoc least significant difference (LSD) tests were used to analyze the differences among multiple groups. The significant difference or correlation among groups was analyzed by two-way ANOVA and/or two-sided paired *t*-test, and Pearson's correlation analysis. Kaplan–Meier curves with the log rank test was used to calculate the overall survival (OS) of CRC patients. All data are presented as mean ± SD.

## 3. Results

### 3.1. ALDH1A3 Upregulation Is Associated with Poor Prognosis of CRC Patients

**Figure 1:**
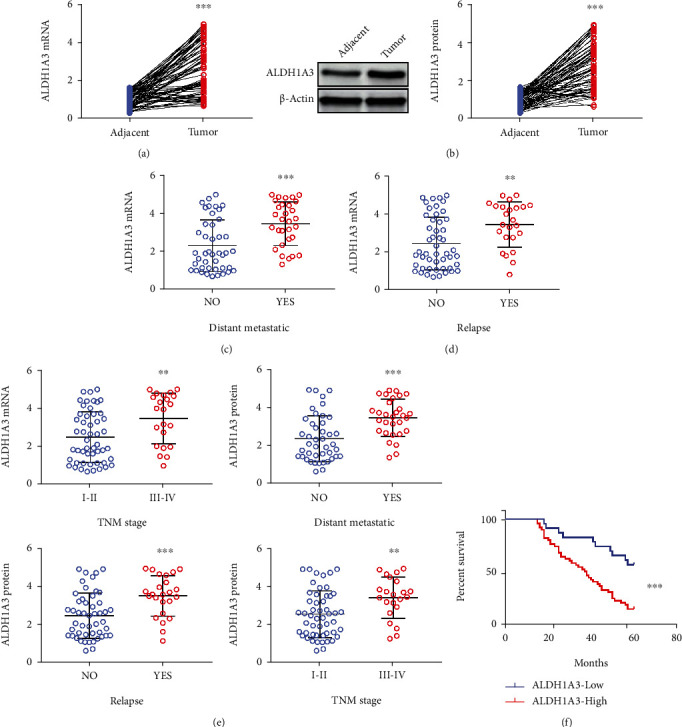
Expression of ALDH1A3 was associated with the clinicopathological characteristics and poor prognosis in patients with colorectal cancer. mRNA (a) and protein level (b) of ALDH1A3 expression in 73 human CRC samples and paired adjacent normal tissues (*n* = 73). The expression level of ALDH1A3 was significantly higher in tumor tissues of (c) patients with metastatic CRC (*n* = 43) than those without metastasis (*n* = 30) or (d) patients with relapse CRC (*n* = 49) compared with those without relapse CRC (*n* = 24) or (e) patients with stage III–IV CRC (*n* = 51) than those with stage I–II CRC (*n* = 22). (f) Kaplan–Meier curve of overall survival.

The expression level of ALDH1A3 in CRC tissues (*n* = 73) was measured by qRT-PCR, in comparison with paired adjacent normal tissues, ALDH1A3 mRNA was significantly increased in tumor samples (Figure 1(a)). Western blot results showed markedly elevated protein level of ALDH1A3 in CRC tissues compared to that in controls (Figure 1(b)). Moreover, ALDH1A3 expression was higher in tumors of patients with metastatic CRC (*n* = 43) than those without metastasis (*n* = 30) (Figure 1(c)). In addition, patients with relapse CRC (*n* = 49) had higher ALDH1A3 expression compared with patients without relapse (*n* = 24) (Figure 1(d)). Besides, higher ALDH1A3 expression positively correlated with the TNM stages of CRC and poor overall survival. ALDH1A3 expression was higher in tumors with higher CRC TNM grade (Figure 1(e)). Patients with higher ALDH1A3 expression in had a shorter survival time compared to patients with lower expression of ALDH1A3 (Figure 1(f)).

### 3.2. Knockdown of ALDH1A3 Represses CRC Proliferation and Metastasis *In Vitro*

**Figure 2:**
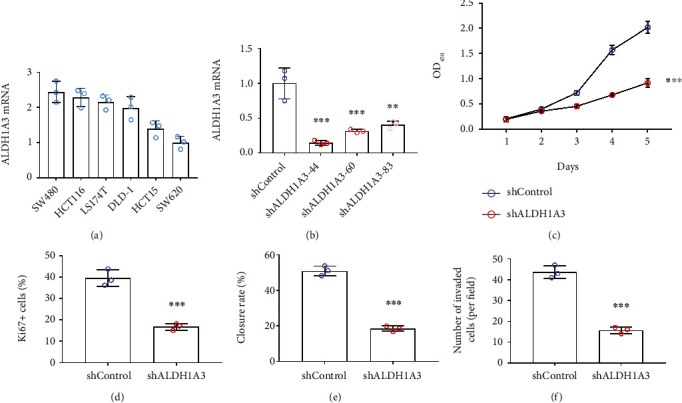
Knockdown of ALDH1A3 repressed CRC cell proliferation, migration, and invasion *in vitro*. (a) ALDH1A3 mRNA level in multiple CRC cell lines. (b) ALDH1A3 silencing efficiency in SW480 cells by shRNAs TRCN0000027144 (shorted to 44), TRCN0000027160 (shorted to 60), and TRCN0000027183 (shorted to 83) was verified by qPCR analysis. (c) The cell viability of ALDH1A3-silenced SW480 cells and the control cells was measured by CCK-8 assay. (d) Proliferation of SW480 cells was analyzed with Ki67 staining by flow cytometry. (e) The migration of ALDH1A3-silenced CRC cells was determined by wound healing assay. (f) The invasion of ALDH1A3-silenced CRC cells was analyzed by trans-well assay (3 wells per group).

To investigate the role of ALDH1A3 in CRC, we examined the mRNA level of ALDH1A3 in six CRC cell lines (SW480, HCT116, LS174T, DLD-1, HCT15, and SW620), and found that ALDH1A3 was highest expressed in SW480 cells, which was used in further studies (Figure 2(a)). ALDH1A3 knockdown SW480 cell line was established through shRNA method. Among three shRNAs, shRNA TRCN0000027144 showed the highest efficacy and was used for silencing ALDH1A3 in following experiments (Figure 2(b)). CCK-8 assay indicated that ALDH1A3 knockdown reduced cell viability of CRC cells significantly (Figure 2(c)). We performed flow cytometry analysis using Ki 67 staining to examine the cancer cell proliferation with and without ALDH1A3. Upon ALDH1A3 knockdown, Ki67 positive SW480 cell was decreased markedly relative to that in control group (Figure 2(d)). In addition, migration and trans-well assays showed that ALDH1A3 silencing decreased the ability of wound healing and invasion significantly in SW480 cells (Figures 2(e) and 2(f)).

### 3.3. ALDH1A3 Mediates the Expression of Linc00284 *In Vitro* and *In Vivo*

**Figure 3:**
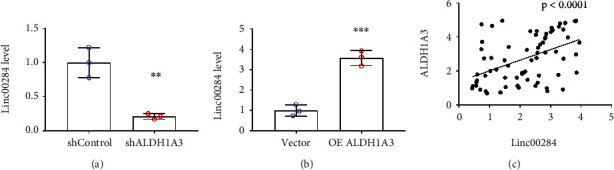
ALDH1A3 mediated the expression of Linc00284 *in vitro* and in CRC tissues of patients. The expression of Linc00284 in ALDH1A3-silenced (a) and -overexpressed (b) SW480 cells was determined by qPCR analysis. (c) The correlation between ALDH1A3 and Linc00284 was analyzed by Pearson correlation test in 73 human CRC samples.

Our previous study indicated that Linc00284 plays an important role in CRC progression [12], qPCR analysis showed that knockdown of ALDH1A3 significantly reduced the expression level of Linc00284 (Figure 3(a)), while overexpression of ALDH1A3 upregulated Linc00284 expression markedly (Figure 3(b)). Interestingly, ALDH1A3 expression was correlated with Linc00284 expression positively (Figure 3(c)). The above data indicated that ALDH1A3 might promote invasion of CRC by regulating the expression level of Linc00284.

### 3.4. ALDH1A3–Linc00284 Axis Promotes EMT via Regulating TGF*β* Level

**Figure 4:**
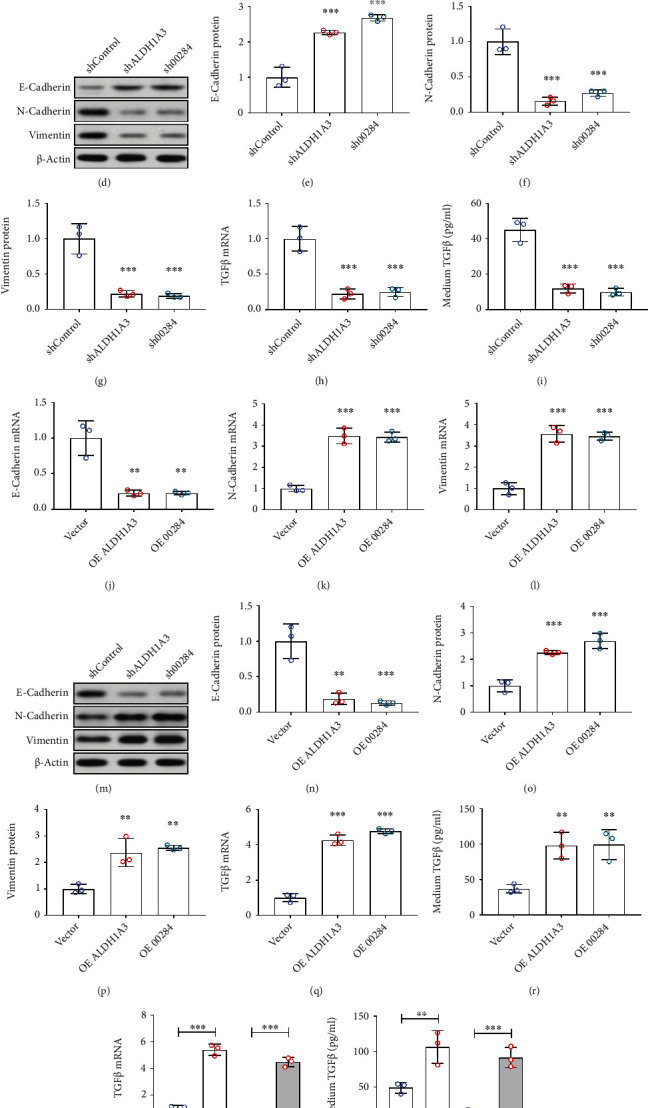
ALDH1A3–Linc00284 axis promoted EMT via regulating TGF*β* level. The mRNA level of E-cadherin (a); N-cadherin (b); and vimentin (c) in Linc00284- and ALDH1A3-silenced SW480 cells was determined by qPCR analysis. The protein level of E-cadherin ((d) and (e)), N-cadherin ((d) and (f)), and vimentin ((d) and (g)) in Linc00284- and ALDH1A3-silenced SW480 cells was determined by western blot analysis. (h) The mRNA level of TGF*β* in Linc00284- and ALDH1A3-silenced SW480 cells was determined by qPCR analysis. (i) The protein level of TGF*β* in Linc00284- and ALDH1A3-silenced SW480 cells medium was determined by ELISA. The mRNA level of E-cadherin (j); N-cadherin (k), and vimentin (l) in Linc00284- and ALDH1A3-overexpressed SW480 cells was determined by qPCR analysis. The protein level of E-Cadherin ((m) and (n)), N-Cadherin ((m) and (o)), and Vimentin ((m) and (p)) in Linc00284- and ALDH1A3-overexpressed SW480 cells was determined by western blot analysis. (q) The mRNA level of TGF*β* in Linc00284- and ALDH1A3-overexpressed SW480 cells was determined by qPCR analysis. (r) The protein level of TGF*β* in Linc00284- and ALDH1A3-overexpressed SW480 cells medium was determined by ELISA. Overexpression of Linc00284 rescued the effect of ALDH1A3 silence on TGF*β* mRNA level in SW480 cell (s) and protein level (t) in medium (3 wells per group).

To explore the mechanism of ALDH1A3–Linc00284 signal regulating CRC metastasis, EMT-related genes were examined by qPCR and Western blots. Knockdown ALDH1A3 or Linc00284 significantly upregulated E-cadherin expression, while downregulated Vimentin and N-cadherin levels in SW480 cells (Figures 4(a)–4(g)). On contrast, overexpression ALDH1A3 or Linc00284 exhibited the opposite effect on the expression of EMT-related genes, as indicated by deceased mRNA and protein expression of E-cadherin, and increased mRNA and protein expression of Vimentin and N-cadherin (Figures 4(j)–4(p)), which suggests the inhibition of EMT process by overexpression of ALDH1A3 or Linc00284. TGF*β* signaling is a key protein factor that mediates the process of EMT [19]. Both qPCR and ELISA data showed that knockdown ALDH1A3 or Linc00284 significantly reduced the expression level of TGF*β*, while overexpression had the opposite effect (Figures 4(h)–4(i) and 4(q)–4(r)). Moreover, overexpression of Linc00284 could reverse the downregulation of ALDH1A3 knockdown on TGF*β* mRNA and protein (Figures 4(s)–4(t)). These results suggested that ALDH1A3–Linc00284 might promote EMT process by regulating TGF*β* signal, and then promote CRC tumor metastasis.

### 3.5. ALDH1A3–Linc00284 Axis Mediates the Expression of TGF*β* by Sponging miR-361-5p

**Figure 5:**
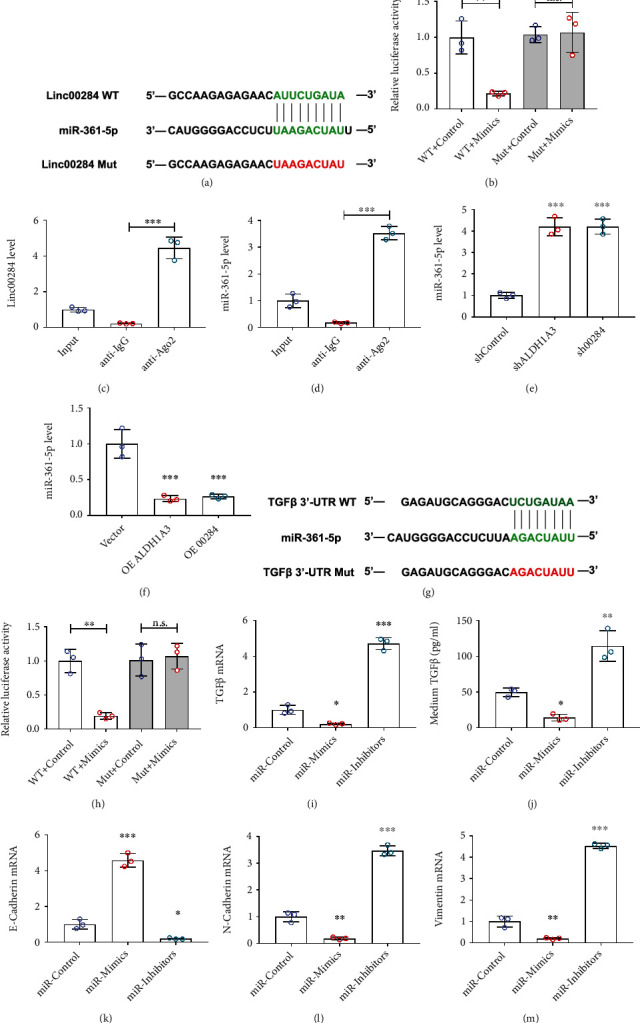
ALDH1A3–Linc00284 axis mediated the expression of TGF*β* by sponging miR-361-5p. (a) Schematic diagram of the potential binding sites between Linc00284 and miR-361-5p. Dual-luciferase reporter assay (b) and RIP experiment (c) and (d) confirmed the interaction between Linc00284 and miR-361-5p. (e) and (f) The expression level of miR-361-5p in ALDH1A3- or Linc00284-silenced (e) and -overexpressed (f) SW480 cells. (g) Schematic diagram of the potential binding sites between 3'-UTR of TGF*β* mRNA and miR-361-5p. (h) Dual-luciferase reporter assay confirmed the interaction between TGF*β* mRNA and miR-361-5p. The mRNA level in cells (i) and protein in medium (j) of TGF*β* in miR-361-5p mimics- or inhibitors-treated SW480 cells were examined by qPCR and ELISA, respectively. The mRNA level of E-Cadherin (k), N-cadherin (l), and Vimentin (m) in miR-361-5p mimics- or inhibitors-treated SW480 cells was examined by qPCR.

We searched the TargetScan database and identified the microRNA candidate that can bind to Linc00284 and TGF*β*. Sequence analysis showed that miR-361-5p has the complementary sequences that match Linc00284 and TGF*β*, respectively (Figures 5(a) and 5(g)). To confirm the interaction between miR-361-5p and Linc00284 or TGF*β*, we constructed the wild type and mutant overexpression plasmids of Linc00284 and TGF*β* (Figures 5(a) and 5(g)), and then performed luciferase assay. The results indicated that miR-361-5p bound to Linc00284 and TGF*β* (3'-UTR) directly (Figures 5(b) and 5(h)), which was further confirmed by RIP assay (Figures 5(c) and 5(d)). Accordingly, miR-361-5p was increased in ALDH1A3- or Linc00284-silenced CRC cells, while decreased in ALDH1A3- or Linc00284-overexpressed CRC cells significantly (Figures 5(e) and 5(f)). As shown in Figures 5(i) and 5(j), miR-361-5p mimics (overexpression) decreased the TGF*β* level, while miR-361-5p inhibitors (knockdown) increased TGF*β* expression significantly. Identical results were observed on the expression of EMT-related genes, E-cadherin (Figure 5(k)), N-cadherin (Figure 5(l)), and Vimentin (Figure 5(m)).

### 3.6. Inhibition of miR-361-5p Rescues the Effect ALDH1A3–Linc00284 on CRC Progression

**Figure 6:**
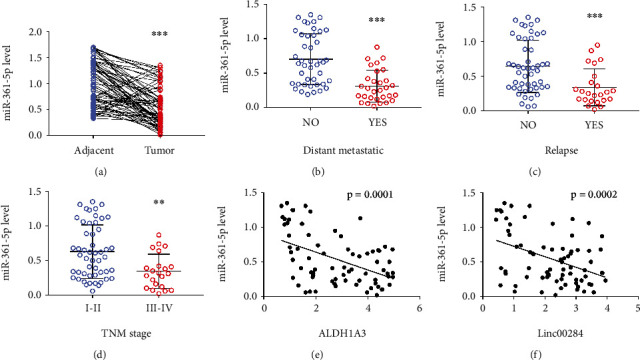
Expression of ALDH1A3–Linc00284-mediated miR-361-5p was associated with the clinicopathological characteristics in patients with colorectal cancer. (a) The expression level of miR-361-5p in 73 human CRC samples and paired adjacent normal tissues (*n* = 73). The expression level of miR-361-5p was significantly lower in tumor tissues of (b) patients with metastatic CRC (*n* = 43) than those without metastasis (*n* = 30) or (c) patients with relapse CRC (*n* = 49) compared with those without relapse CRC (*n* = 24) or (d) patients with stage III–IV CRC (*n* = 51) than those with stage I–II CRC (*n* = 22). The correlation between miR-361-5p and ALDH1A3 (e) and Linc00284 (f) was analyzed by the Pearson correlation test in 73 human CRC samples.

**Figure 7:**
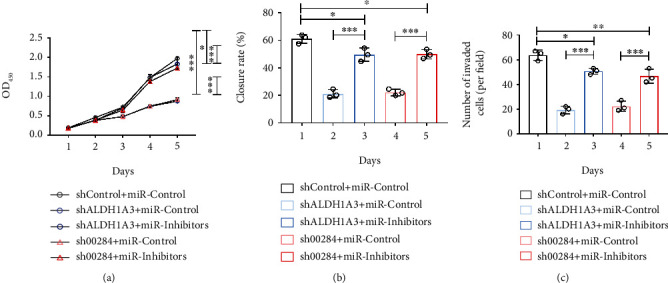
Inhibition of miR-361-5p rescued the effect ALDH1A3 or Linc00284 knockdown in CRC cells. The effect of miR-361-5p treatment on cell viability (a), migration (b), and invasion (c) of ALDH1A3- or Linc00284-silenced SW480 cells was evaluated by CCK-8 assay, wound healing assay, and trans-well assay, respectively (3 wells per group).

In human CRC tissues, the expression of miR-361-5p was decreased significantly relative to that in adjacent normal tissues (Figure 6(a)). Interestingly, miR-361-5p was reduced in distant metastasis (Figure 6(b)), relapse (Figure 6(c)), and high TNM staging CRC (Figure 6(d)). Furthermore, miR-361-5p expression negatively correlated with the expression of both ALDH1A3 and Linc00284 in CRC tissues (Figures 6(e) and 6(f)). Next, we silenced miR-361-5p in ALDH1A3- or Linc00284-knockdown SW480 cells and examined the proliferation and metastasis of CRC cells. As expected, inhibition of miR-361-5p reversed the effects of ALDH1A3- or Linc00284-knockdown on cell viability (Figure 7(a)). Identical effects of miR-361-5p inhibition on CRC cell migration and invasion were observed by wound healing assay and trans-well assay (Figures 7(b) and 7(c)). The above data further indicated that ALDH1A3-Linc00284 mediates CRC invasion through regulating the expression miR-361-5p.

## 4. Discussion

Long noncoding RNAs play an important role in the tumor initiation and progression [11, 20–24]. Multiple studies showed higher Linc00284 expression in breast cancer and liver cancer, which can promote the tumor progression and associated with poor overall survival [11, 25, 26]. Our recent study demonstrated that the upregulation of Linc00284 in tumor samples of CRC patients; in addition, Linc00284 expression positively correlated with metastasis, recurrence, and poor survival [12]. However, the underlying mechanism of Linc00284-mediated CRC progression is still unclear. In this study, we revealed that Linc00284 was upregulated by ALDH1A3 in CRC tissues and cells, and the ALDH1A3–Linc00284 axis promoted the invasion of CRC through activation of TGF*β* signaling and downstream EMT process. Mechanistically, upregulation of ALDH1A3–Linc00284 promotes colorectal cancer invasion and migration by regulating the miR-361-5p/TGF*β* signaling pathway, which might provide a new insight into the treatment of CRC.

EMT not only plays a critical role in tumor stemness, proliferation, migration, and invasion but also associates with the tumor microenvironment to induce immunosuppression and cause resistance to therapy [27, 28]. TGF*β* signaling can trigger EMT when cells are in the certain disease microenvironment, such as fibrosis and cancer [28, 29]. In CRC, TGF*β* and downstream factors are mobilized by integrin, which promotes the EMT processed in both cell and animal models [30]. On the contrary, decreasing TGF*β* expression and activity in the tumor microenvironment leads to potent immune responses of CRC in rodent models [31, 32]. In line with the above findings, knockdown ALDH1A3–Linc00284 axis significantly affects the expression level of EMT-related genes E-cadherin, N-cadherin, and Vimentin, and inhibits the EMT process, while overexpression of each of them has opposite effects. Moreover, changing the expression levels of the ALDH1A3–Linc00284 axis also affects the viability and invasion of CRC cells. Importantly, we found that higher expression of ALDH1A3–Linc00284 positively correlates with the TNM staging and poor survival of CRC patients. These findings suggested that ALDH1A3–Linc00284 promotes the EMT process by regulating the TGF*β* signal, and then promotes CRC tumor metastasis.

microRNAs are small noncoding RNAs that function as either tumor suppressors or oncogenes under certain conditions [33]. More and more studies have identified miRNAs as potential biomarkers for human cancer diagnosis, prognosis and treatment targets. The miR-361-5p has been reported widely expressed in many tissues. Higher expression of miR-361-5p indicates better prognosis of breast cancer patients [34]. In gastric cancer, miR-361-5p can suppress chemoresistance of SGC-7901 and MKN-28 cells through inhibition of the expression of FOXM1 [35]. Here, we identified that TGF*β* is a direct target of miR-361-5p in CRC cells, silencing miR-361-5p can induce the expression of TGF*β* and promote the EMT process. Interestingly, sequence analysis revealed that miR-361-5p can be bound by Linc00284 in SW480 cells. In tumor samples of CRC patients, miR-361-5p expression is negatively correlated with the expression of both ALDH1A3 and Linc00284. More importantly, inhibition of miR-361-5p can rescue the effect ALDH1A3 or Linc00284 knockdown in CRC cells. Our findings indicate that the ALDH1A3–Linc00284 axis mediates the progression of CRC by targeting TGF*β* signaling via sponging miR-361-5p. Animal models need to be established to further investigate and confirm the regulatory effect of ALDH1A3–Linc00284-miR-361-5p in the CRC tumor microenvironment in the future studies.

## 5. Conclusions

Our findings indicate that the ALDH1A3–Linc00284 axis mediates the progression of CRC by targeting TGF*β* signaling via sponging miR-361-5p in CRC cells, providing new insight into the pathogenesis and treatment of colorectal cancer.

## Figures and Tables

**Table 1:** Primer used in this study.

	Forward primer	Reverse primer
*Linc00284*	5'-CCAGGGGATAAAACCCGCTT-3'	5'-TAAGCACCAAGTCACGCTGT-3'
*U6*	5'-CTCGCTTCGGCAGCACA-3'	5'-AACGCTTCACGAATTTGCGT-3'
*GAPDH*	5'-TCAACGACCACTTTGTCAAGCTCA-3'	5'-GCTGGTGGTCCAGGGGTCTTACT-3'
*miR-361-5p*	5'-ATAAAGRGCRGACAGTGCAGATAGTG-3'	5'-TCAAGTACCCACAGTGCGGT-3'
*ALDH1A3*	5'-TCTCGACAAAGCCCTGAAGT-3'	5'-TATTCGGCCAAAGCGTATTC-3'
*TGFβ*	5'-GGCCAGATCCTGTCCAAGC-3'	5'-GTGGGTTTCCACCATTAGCAC-3'
*Vimentin*	5'-GCCCTAGACGAACTGGGTC-3'	5'-GGCTGCAACTGCCTAATGAG-3'
*E-cadherin*	5'-CGAGAGCTACACGTTCACGG-3'	5'-GGGTGTCGAGGGAAAAATAGG-3'
*N-cadherin*	5'-TCAGGCGTCTGTAGAGGCTT-3'	5'-ATGCACATCCTTCGATAAGACTG-3'

## Data Availability

The datasets used and/or analyzed during the current study are available from the corresponding author upon reasonable request.

## References

[1] Miao X., Li Z., Zhang Y., Wang T. (2021). MicroRNA-4284 inhibits colon cancer epithelial-mesenchymal transition by down-regulating Perilipin 5. *STEMedicine*.

[2] Siegel R. L., Miller K. D., Goding Sauer A. (2020). Colorectal cancer statistics, 2020. *CA Cancer Journal for Clinicians*.

[3] Akimoto N., Ugai T., Zhong R. (2021). Rising incidence of early-onset colorectal cancer -- a call to action. *Nature Reviews. Clinical Oncology*.

[4] Saad El Din K., Loree J. M., Sayre E. C. (2020). Trends in the epidemiology of young-onset colorectal cancer: a worldwide systematic review. *BMC Cancer*.

[5] Shinagawa T., Tanaka T., Nozawa H. (2018). Comparison of the guidelines for colorectal cancer in Japan, the USA and Europe. *Annals of Gastroenterological Surgery*.

[6] Murphy N., Ward H. A., Jenab M. (2019). Heterogeneity of colorectal cancer risk factors by anatomical subsite in 10 European countries: a multinational cohort study. *Clinical Gastroenterology and Hepatology*.

[7] Ganesh K., Stadler Z. K., Cercek A. (2019). Immunotherapy in colorectal cancer: rationale, challenges and potential. *Nature Reviews. Gastroenterology & Hepatology*.

[8] Strous M. T. A., Janssen-Heijnen M. L. G., Vogelaar F. J. (2019). Impact of therapeutic delay in colorectal cancer on overall survival and cancer recurrence—is there a safe timeframe for prehabilitation?. *European Journal of Surgical Oncology*.

[9] Durinikova E., Kozovska Z., Poturnajova M. (2018). ALDH1A3 upregulation and spontaneous metastasis formation is associated with acquired chemoresistance in colorectal cancer cells. *BMC Cancer*.

[10] Feng H., Liu Y., Bian X., Zhou F., Liu Y. (2018). ALDH1A3 affects colon cancer _in vitro_ proliferation and invasion depending on CXCR4 status. *British Journal of Cancer*.

[11] Vidovic D., Huynh T. T., Konda P. (2020). ALDH1A3-regulated long non-coding RNA NRAD1 is a potential novel target for triple-negative breast tumors and cancer stem cells. *Cell Death and Differentiation*.

[12] You J., Li J., Ke C. (2021). Oncogenic long intervening noncoding RNA Linc00284 promotes c-Met expression by sponging miR-27a in colorectal cancer. *Oncogene*.

[13] Thomas M. L., de Antueno R., Coyle K. M. (2016). Citral reduces breast tumor growth by inhibiting the cancer stem cell marker ALDH1A3. *Molecular Oncology*.

[14] Ruan Z., Zhao D. (2019). Long intergenic noncoding RNA LINC00284 knockdown reduces angiogenesis in ovarian cancer cells via up-regulation of MEST through NF-*κ*B1. *The FASEB Journal*.

[15] Sheng W., Guo W., Lu F., Liu H., Xia R., Dong F. (2021). Upregulation of Linc00284 promotes lung cancer progression by regulating the miR-205-3p/c-Met axis. *Frontiers in Genetics*.

[16] Wang S., Zhang L., Tao L. (2020). Construction and investigation of an LINC00284-associated regulatory network in serous ovarian carcinoma. *Disease Markers*.

[17] Zhou B., Ge Y., Shao Q., Yang L., Chen X., Jiang G. (2021). Long noncoding RNA LINC00284 facilitates cell proliferation in papillary thyroid cancer via impairing miR-3127-5p targeted E2F7 suppression. *Cell Death Discovery*.

[18] Kim K. H., Sederstrom J. M. (2015). Assaying cell cycle status using flow cytometry. *Current Protocols in Molecular Biology*.

[19] Xu J., Lamouille S., Derynck R. (2009). TGF-*β*-induced epithelial to mesenchymal transition. *Cell Research*.

[20] Chen S., Liang H., Yang H. (2017). LincRNa-p21: function and mechanism in cancer. *Medical Oncology*.

[21] Shen P., Pichler M., Chen M., Calin G. A., Ling H. (2017). To Wnt or lose: the missing non-coding Linc in colorectal cancer. *International Journal of Molecular Sciences*.

[22] Ulitsky I., Bartel D. P. (2013). lincRNAs: genomics, evolution, and mechanisms. *Cell*.

[23] Wu T., Huang C., Wang F. (2022). LncRNA DLX6-AS1 regulates osteosarcoma progression via the miR-200a-3p/GPM6P axis. *STEMedicine*.

[24] Zhou J., Liu J., Ma W., Zhao P. (2022). Hsa_circ_0002111/miR-557/DUSP14 axis mediates euthyrox-resistance in papillary thyroid cancer. *STEMedicine*.

[25] Xu J., Zhang J., Shan F., Wen J., Wang Y. (2019). SSTR5-AS1 functions as a ceRNA to regulate CA2 by sponging miR-15b-5p for the development and prognosis of HBV-related hepatocellular carcinoma. *Molecular Medicine Reports*.

[26] Zhao Y., Wang H., Wu C. (2018). Construction and investigation of lncRNA-associated ceRNA regulatory network in papillary thyroid cancer. *Oncology Reports*.

[27] Horn L. A., Fousek K., Palena C. (2020). Tumor plasticity and resistance to immunotherapy. *Cancer*.

[28] Yang J., Antin P., Berx G. (2020). Guidelines and definitions for research on epithelial-mesenchymal transition. *Nature Reviews. Molecular Cell Biology*.

[29] Nieto M. A., Huang R. Y., Jackson R. A., Thiery J. P. (2016). Emt: 2016. *Cell*.

[30] Bates R. C., Bellovin D. I., Brown C. (2005). Transcriptional activation of integrin beta6 during the epithelial-mesenchymal transition defines a novel prognostic indicator of aggressive colon carcinoma. *The Journal of Clinical Investigation*.

[31] Brown N. F., Marshall J. F. (2019). Integrin-mediated TGF*β* activation modulates the tumour microenvironment. *Cancers*.

[32] Rachidi S., Metelli A., Riesenberg B. (2017). Platelets subvert T cell immunity against cancer via GARP-TGF*β* axis. *Science Immunology*.

[33] Peng Y., Croce C. M. (2016). The role of MicroRNAs in human cancer. *Signal Transduction and Targeted Therapy*.

[34] Cao Z. G., Huang Y. N., Yao L. (2016). Positive expression of miR-361-5p indicates better prognosis for breast cancer patients. *Journal of Thoracic Disease*.

[35] Tian L., Zhao Z., Xie L., Zhu J. (2018). MiR-361-5p suppresses chemoresistance of gastric cancer cells by targeting FOXM1 via the PI3K/Akt/mTOR pathway. *Oncotarget*.

